# Pediatric diencephalic tumors: a constellation of entities and management modalities

**DOI:** 10.3389/fonc.2023.1180267

**Published:** 2023-07-13

**Authors:** Soniya N. Pinto, Jason Chiang, Ibrahim Qaddoumi, David Livingston, Asim Bag

**Affiliations:** ^1^Department of Diagnostic Imaging, St. Jude Children’s Research Hospital, Memphis, TN, United States; ^2^Department of Pathology, St. Jude Children’s Research Hospital, Memphis, TN, United States; ^3^Departments of Global Pediatric Medicine, St. Jude Children’s Research Hospital, Memphis, TN, United States; ^4^Department of Radiology, University of Tennessee Health Science Center, Memphis, TN, United States

**Keywords:** adenoma, craniopharyngioma, diencephalon, germ cell tumor, langerhans cell histiocytosis, pineoblastoma

## Abstract

The diencephalon is a complex midline structure consisting of the hypothalamus, neurohypophysis, subthalamus, thalamus, epithalamus, and pineal body. Tumors arising from each of these diencephalic components differ significantly in terms of biology and prognosis. The aim of this comprehensive review is to describe the epidemiology, clinical symptoms, imaging, histology, and molecular markers in the context of the *2021 WHO classification of central nervous system neoplasms*. We will also discuss the current management of each of these tumors.

## 1 Introduction

The diencephalon, an area in the central part of the brain that encompasses crucial structures including the thalamus and hypothalamus, is a relatively uncommon site for primary pediatric brain tumors. Yet, when tumors do occur in this location, they present unique challenges in neuro-oncology due to their intricate location and diverse clinical manifestations (1).

This review article aims to provide a comprehensive overview of diencephalic tumors, shedding light on their epidemiology, clinical presentation, imaging, and histopathology and the current strategies employed in their management. We explore the unique neuroanatomical considerations and the wide-ranging symptomatology that these tumors can exhibit. Additionally, we delve into the molecular genetics underlying these neoplasms, recognizing the increasing role of molecular profiling in the classification and treatment of brain tumors. We further discuss the current standards and innovations in the diagnosis and management of these tumors, including neuroimaging techniques, surgical approaches, radiation therapy, and emerging targeted therapies. Acknowledging the multifaceted challenges posed by diencephalic tumors, this review also aims to highlight the existing gaps in our understanding and emphasize areas where further research is required. Through a comprehensive exploration of diencephalic tumors, this review hopes to provide insights into this complex domain, paving the way for improved patient outcomes.

The diencephalon is embryologically derived from the prosencephalon, which evolves to develop the epithalamus superiorly, the thalamus centrally, and the subthalamus and hypothalamus inferiorly (1). The epithalamus comprises the anterior and posterior paraventricular nuclei, the habenular nuclei and their interconnecting fibers, the habenular commissure, the stria medullaris, and the pineal body. The epithalamus connects the limbic system to the other parts of the brain and is responsible for melatonin secretion. The thalamus is the largest part of the diencephalon that surrounds the third ventricle and is subdivided into multiple nuclei that function as relay centers for sensory and motor information to and from the cerebral cortex. The subthalamus is a complex structure that comprises a group of subthalamic nuclei and tracts that mediate fine control of motor movement. The hypothalamus is located inferior and anterior to the thalamus, forming the inferolateral walls of the third ventricle. It consists of a group of nuclei that coordinate with the autonomic and endocrine system to control metabolism, energy balance, reproduction, thermoregulation, fluid and electrolyte balance, and immune and emotional responses. The hypothalamus is intimately connected with the neurohypophysis (posterior pituitary).

As diencephalic structures mediate important functions, tumors of this region often have widespread and lasting implications on neurological and other metabolic functions, particularly in the pediatric population (2). Surgical resection of tumors in this region is often technically challenging and fraught with morbidity (3). Therefore, clinical management requires a comprehensive approach with an astute recognition and knowledge of clinical symptoms, laboratory markers, cross-sectional imaging, histologic and molecular pathology, detailed surgical planning, radiation therapy, chemotherapy, and targeted molecular therapy.

Tumors arising from each diencephalic component differ histologically and genetically despite their common embryological origin. Based on tropism, diencephalic tumors can be broadly classified into four groups, hypothalamic tumors, tumors of the neurohypophysis, thalamic tumors, and tumors of the pineal region, and we will systematically approach tumors arising from these four groups.

## 2 Hypothalamic tumors

Hypothalamic tumors may arise from either the neuronal or glial components of the hypothalamus or may invade the hypothalamus from adjacent structures (**Table 1**) (5).

**Table 1:** Primary and infiltrating neoplasms of the hypothalamus in pediatric patients (4).

Primary site of origin	Cell of origin	Tumor
Hypothalamus	Glial cell	Hypothalamic/optic pathway glioma
		Glioblastoma multiforme, *IDH*-mutant
		Chordoid glioma
	Neuronal cell	Extraventricular neurocytoma
		Gangliocytoma
		Gangliogliomas
	Glial and neuronal cells	Hamartoma
Adenohypophysis	Anterior pituitary cells	Pituitary adenoma
	Cellular elements of Rathke’s pouch	Craniopharyngioma
Meninges	Meningothelial cells of the arachnoid	Meningioma
Clivus	Notochordal remnants	Chordoma
Neurohypophysis	Monocytes, macrophages, and dendritic cells	Langerhans cell histiocytosis
		Germ cell tumor
	Glial cell	Pituicytomas
	Neuronal cell	Gangliocytomas
		Neurocytomas

### 2.1 Primary hypothalamic tumors

#### 2.1.1 Glial tumors

##### 2.1.1.1 Hypothalamic/optic pathway gliomas

Gliomas are the most common primary tumors arising from the hypothalamus in the pediatric population and are usually low grade and slow growing. These tumors are related to either *BRAF* or *NF1* aberrations. They can be limited to the optic pathway with secondary involvement of the hypothalamus or can involve both optic pathway and hypothalamus. Gliomas limited to the hypothalamus without any optic pathway involvement are possible but rare (5).

###### 2.1.1.1.1 Clinical symptoms

Hypothalamic tumors clinically present with visual disturbances and endocrine dysfunctions. In children under the age of 3, this tumor may present with a unique diencephalic syndrome characterized by failure to thrive and emaciation despite normal caloric intake. Locomotor hyperactivity and increased alertness may also occur (6).

###### 2.1.1.1.2 Imaging

**Figure 1:**
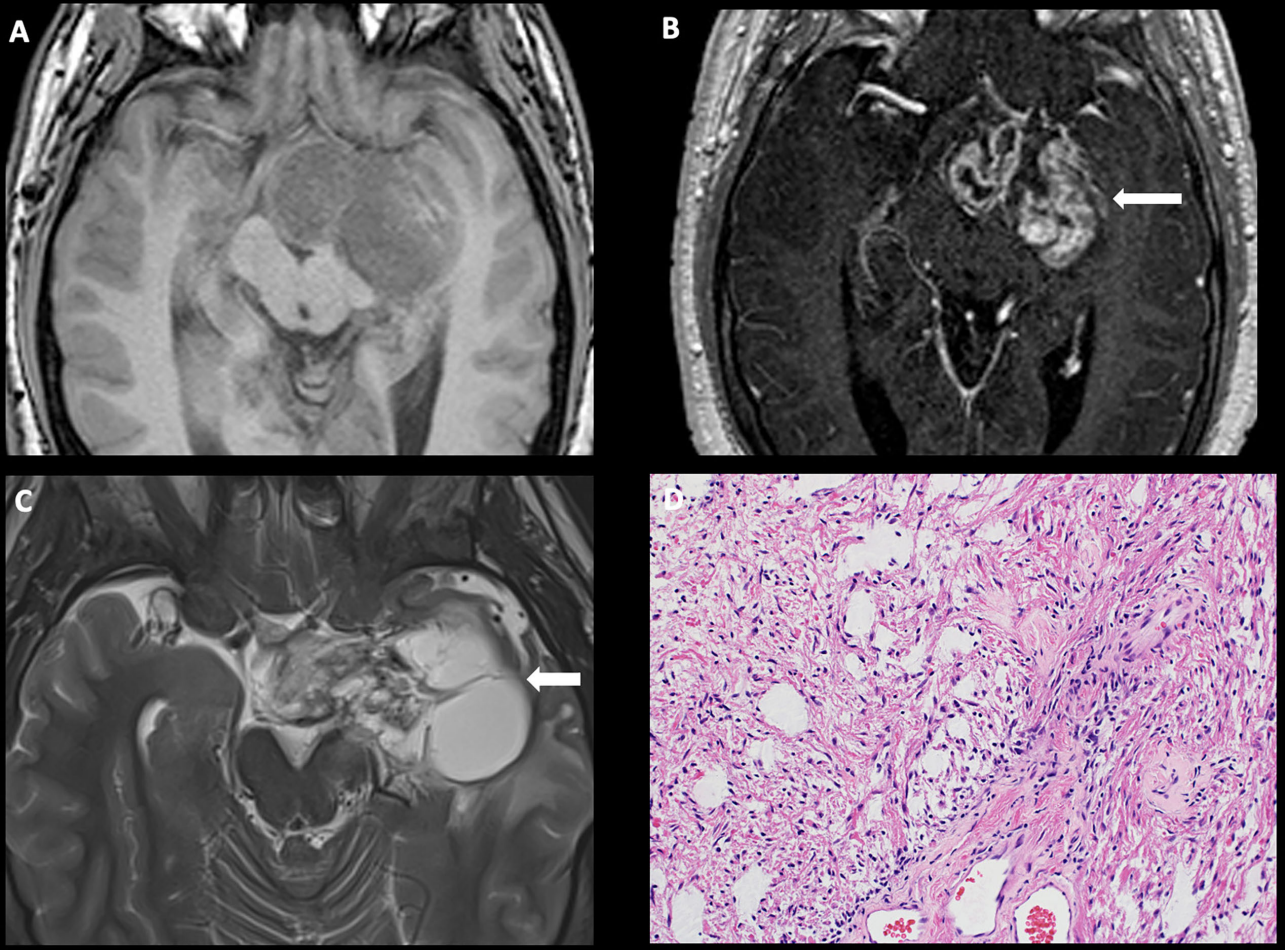
Obtained from a patient at St. Jude Children’s Research Hospital, Memphis, Tennessee. Axial pre-contrast T1-weighted **(A)** and post-contrast T1-weighted **(B)** images, demonstrating a complex mass centered in the left greater than right hypothalamus/optic chiasm, with extension into the left temporal lobe and enhancing solid components (arrow). **(C)** Axial T2-weighted sequence demonstrating a large cyst in the left temporal lobe (arrow). The histologic section **(D)** demonstrates a glial neoplasm with alternating loose and more compact architecture and low levels of mitotic activity. Many eosinophilic Rosenthal fibers are present in the background. Hyalinized vessels are typical findings.

These tumors typically demonstrate intrinsic T1 isointensity to hypointensity and T2/FLAIR hyperintensity. These tumors can be entirely solid or purely cystic or have mixed solid and cystic components. They can also be either well circumscribed or diffusely infiltrative, particularly if the tumors are associated with neurofibromatosis-1 (NF1). The enhancement pattern of these tumors is extremely variable. The solid component can demonstrate no enhancement, patchy mild enhancement, or intense enhancement. Similarly, the cyst wall can be enhancing or non-enhancing. Diffusion restriction is characteristically absent in these tumors (7). Extension along the cisternal, canalicular, and orbital segments of the optic nerves and the optic tracts is frequently seen (**Figure 1**) (8). The infiltrative tumors can involve bilateral medial temporal lobes and basal ganglia.

###### 2.1.1.1.3 Histology and molecular markers

It was found that 50%–70% of hypothalamic/optic pathway gliomas in the pediatric population are associated with NF1, an autosomal dominant disorder characterized by loss-of-function mutation in the *NF1* gene, a negative regulator of the mitogen-activated protein kinase (MAPK) pathway (9). Most patients with NF1-associated hypothalamic/optic pathway gliomas arise in children younger than 7 years and may regress spontaneously without treatment (10). In contrast, sporadic hypothalamic/optic pathway gliomas (i.e., those not associated with NF1) arise throughout childhood, are more likely to progress, cause visual impairment, and require therapeutic intervention (11, 12). The majority of these tumors are WHO grade 1 pilocytic astrocytomas, with a few tumors demonstrating the more cellular pilomyxoid histology. The most common molecular aberration in these tumors is the activation of the *BRAF* oncogene, either via a *KIAA1549::BRAF* fusion or a *BRAF* p.V600E mutation, which in turn phosphorylates downstream targets MEK1 and MEK2 of the MAPK pathway (13). Compared to the posterior fossa pilocytic astrocytomas, the incidence of *BRAF* p.V600E point mutation is higher in hypothalamic/optic pathway tumors (14).

###### 2.1.1.1.4 Management

MRI examination plays a central role in the diagnosis, assessment of extent, and monitoring of the response to therapy in patients with sporadic hypothalamic/optic pathway gliomas and surveillance in NF1-associated gliomas. Because the imaging appearance of these tumors is diagnostic, surgical biopsy is usually reserved for treatment-resistant cases in which a more detailed histopathologic and molecular analysis is warranted. These biopsies are usually performed via an open craniotomy with a transcallosal approach (15, 16). Complete surgical resection of these tumors without significant morbidity is not technically feasible, with chemotherapy remaining the mainstay of treatment (16). The most commonly used first-line chemotherapeutic regimens include carboplatin and vincristine (CV) combination therapy or thioguanine, procarbazine, lomustine, and vincristine (TPCV) combination therapy or vinblastine monotherapy (17). Depending on what first-line regimen was used (e.g., CV), patients can be switched to alternative regimens (e.g., vinblastine or TPCV) in cases of recurrent or progressive disease (18–20). Although radiotherapy is effective and has higher vision salvage rates, it is often avoided in young children due to a significant risk of intellectual disability, endocrinopathies, ototoxicity, and secondary malignant neoplasms (21). Molecular analysis of hypothalamic/optic pathway gliomas have helped identify valuable targets for therapy, specifically in patients with recurrent/progressive disease. Patients with fusions or duplications of *BRAF* often respond favorably to therapy with MEK inhibitors such as selumetinib and trametinib. On the other hand, point mutations in *BRAF*V600E represent a target for therapy with *BRAF* inhibitors such as vemurafenib and dabrafenib. In tumors with *BRAF*V600E mutations that progress on *BRAF* inhibitors or develop therapy-related toxicity, the addition of a MEK inhibitor often proves efficacious in disease control and enhancing tolerability (22, 23).

##### 2.1.1.2 Chordoid glioma

Chordoid glioma is a rare, well-circumscribed, low-grade glioma characterized by *PRKCA* mutation that typically arises in the anterior third ventricle. Although this is typically an adult tumor (median age at presentation ~45 years), it is occasionally seen in the pediatric population. Pediatric chordoid glioma patients reported in the literature ranged from 7 to 13 years of age.

###### 2.1.1.2.1 Clinical symptoms

Common presentations include obstructive hydrocephalus with symptoms of increased intracranial pressure. Endocrine abnormalities from mass effect on the hypothalamic nuclei and visual disturbances from mass effect to the optic pathway are other common presenting symptoms.

###### 2.1.1.2.2 Imaging

These tumors are hyperdense on non-contrast computed tomography and usually well circumscribed, multilobulated, and enhance avidly with contrast administration. Connection to the hypothalamus can be seen in some tumors, suggesting the site of origin (24).

###### 2.1.1.2.3 Histology and molecular markers

These tumors are WHO grade 2 neoplasms with well-defined borders without evidence of infiltration. Characteristic histology includes clusters and cords of epithelioid cells in a mucinous stroma (25–27). Mutation of the p.D463H in the *PRKCA* gene is now known to be a ubiquitous feature of chordoid gliomas.

###### 2.1.1.2.4 Management

Gross-total resection is the treatment of choice, and a trans-lamina terminalis approach is associated with decreased overall postoperative morbidity as compared with the transcortical and transcallosal approaches. Radiation therapy is considered in patients with subtotal resection, on a case-by-case basis, but the benefit is not well established (27). Prognostic factors are not well elucidated due to rarity of this lesion.

##### 2.1.1.3 High-grade glioma

High-grade gliomas arising from the hypothalamic regions are rare. To date, only one case of glioblastoma multiforme involving the hypothalamus and the optic chiasm has been reported in a pediatric patient who presented with progressive vision loss. On MRI, the lesion was T1 isointense, T2 hyperintense, with marked peripheral enhancement and central necrosis. The patient was treated with subtotal resection, and histopathology confirmed a WHO grade IV neoplasm with anaplastic hyperchromatic cells, atypical mitoses, and vascular proliferation (28).

#### 2.1.2 Glioneuronal and neuronal tumors

##### 2.1.2.1 Ganglioglioma

Gangliogliomas are common pediatric tumors that typically arise from the temporal lobe. However, there are many reports of gangliogliomas that primarily arise from the hypothalamus or secondarily involve the hypothalamus. The imaging characteristics and histopathology are similar to the gangliogliomas arising from elsewhere in the brain and are enumerated in detail in the thalamic tumor group.

##### 2.1.2.2 Neuronal tumors

Neuronal tumors of the hypothalamus are relatively rare in the pediatric population. To date, there has been only one case report of a hypothalamic extraventricular neurocytoma in a child who presented with bitemporal hemianopsia. MRI revealed a circumscribed, multilobulated, avidly enhancing mass in the suprasellar region, and histopathology demonstrated homogeneous cells with a clear cytoplasm, a low mitotic index, and positive staining for synaptophysin and chromogranin A. The patient was treated with subtotal resection via a right frontotemporal approach and completed postoperative intensity-modulated radiation therapy (i.e., 30 Gy in 15 fractions) (29). Although molecular testing was not performed at the time, the *FGFR1*::TACC1 fusion is now known to be highly frequent in extraventricular neurocytomas; a few other *FGFR* alterations have also been identified (4).

### 2.2 Infiltrating tumors of the hypothalamus

#### 2.2.1 Craniopharyngiomas

In the pediatric population, craniopharyngiomas are almost exclusively of adamantinomatous subtype (aCP), with papillary cases very rarely reported. Craniopharyngiomas arise from cellular elements related to the Rathke’s pouch (craniopharyngeal duct), which express oncogenic β-catenin during early embryonic development (30). The typical age of presentation is between 5 and 15 years without a definite sex predilection (31).

##### 2.2.1.1 Clinical symptoms

Craniopharyngiomas usually present with symptoms of increased intracranial pressure such as headache, visual impairment, and endocrine deficits affecting the growth hormone (GH), luteinizing hormone (LH), follicle-stimulating hormone (FSH), thyroid-stimulating hormone (TSH), adrenocorticotropic hormone (ACTH), or antidiuretic hormone (ADH). Almost half of the patients develop a hypothalamic syndrome characterized by morbid obesity, cognitive impairment, personality changes, and psychiatric symptoms either due to primary involvement of the hypothalamus by the mass or secondary to treatment-related complications (32).

##### 2.2.1.2 Imaging

**Figure 2:**
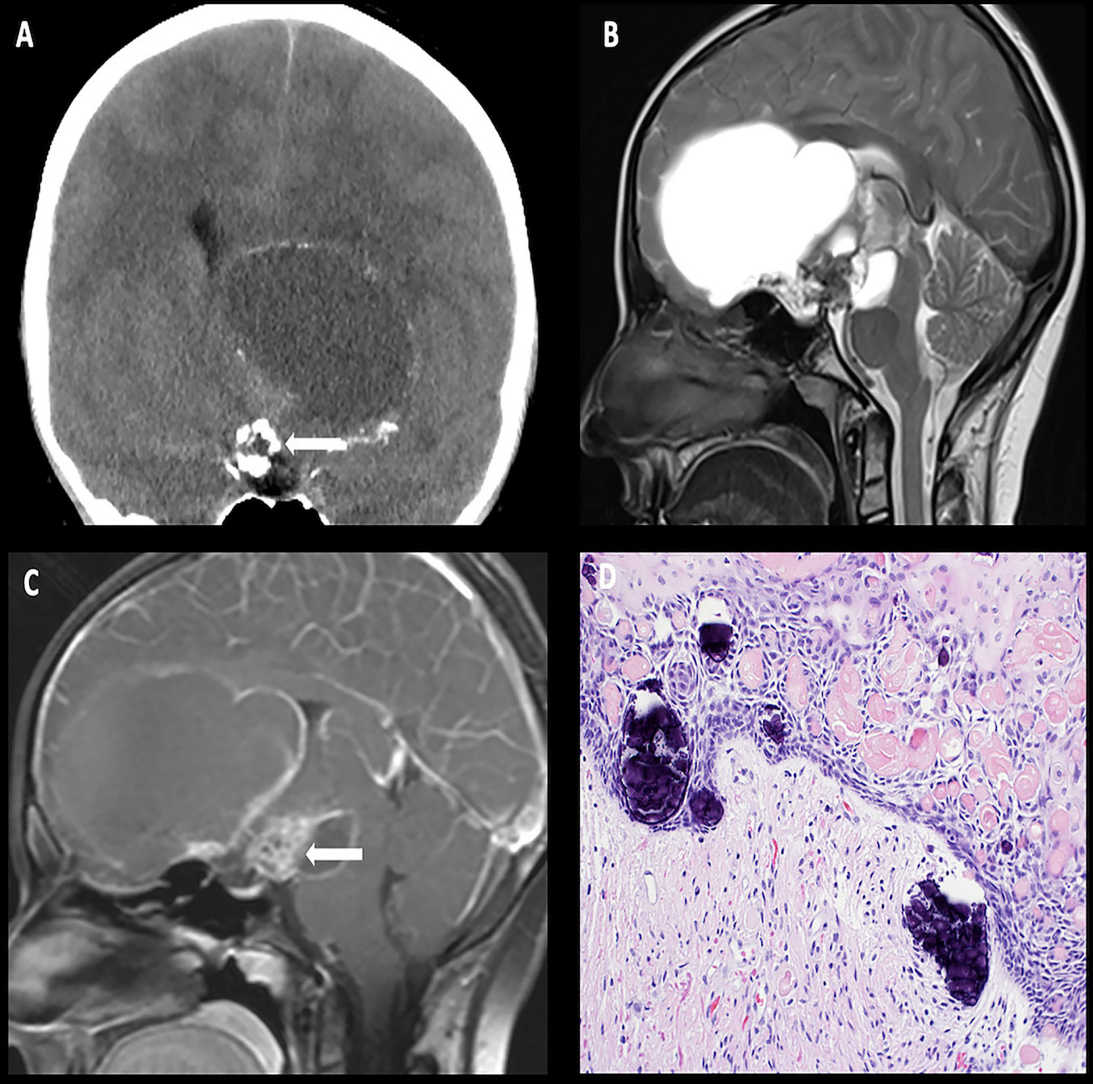
Obtained from a patient at St. Jude Children’s Research Hospital, Memphis, Tennessee. Coronal non-contrast head CT **(A)** demonstrating a calcified solid suprasellar mass (arrow), with a large cystic component extending into the third ventricle and the inferior frontal lobe on the left. Sagittal T2-weighted **(B)** and post-contrast T1-weighted **(C)** images demonstrating enhancing solid components (arrow). The histologic section **(D)** demonstrates an epithelial neoplasm with numerous fragments of “wet keratin” and microcalcifications in the background. The adjacent brain parenchyma shows reactive gliosis. Many Rosenthal fibers are present. Such reactive brain tissue may masquerade as pilocytic astrocytoma.

On imaging, craniopharyngiomas are coarsely calcified, usually cystic, heterogeneously enhancing neoplasms of the sellar, suprasellar, and parasellar regions. The cysts often demonstrate intrinsic T1 hyperintensity/T2 hypointensity due to proteinaceous debris (**Figure 2**) (33). aCPs follow a 90% rule: 90% are cystic, 90% demonstrate calcification, and 90% demonstrate contrast enhancement (34).

##### 2.2.1.3 Histopathology and molecular markers

Histopathologic analysis has demonstrated well-differentiated tumor epithelium-forming cords, lobules, ribbons, nodular whorls, and irregular trabeculae, with degenerative features, such as fibrosis and calcification being commonly found. Occasionally, a xanthogranulomatous reaction to ruptured cyst material may be seen characterized by cholesterol clefts, hemosiderin deposits, xanthoma cells, and multinucleated giant cells. On molecular testing, demonstration of the *CTNNB1* mutation and the absence of *BRAF* p.V600E may be helpful (4).

##### 2.2.1.4 Management

The tumor’s imaging appearance is so characteristic that treatment usually proceeds without a surgical biopsy (34). The cystic components can often enlarge and cause symptoms secondary to mass effect; therefore, stereotactic cyst decompression with Ommaya catheter placement is often a first-line approach. The solid components of the tumor can usually be resected via an open transcranial technique or via newer minimally invasive techniques, such as the transsphenoidal, endoscopic endonasal, or supraorbital approaches (35). Although gross total resection is associated with a lower recurrence rate, it is also associated with significant hypothalamic and visual morbidity (30). Furthermore, many studies do not support a survival advantage of gross total resection over subtotal resection followed by adjuvant radiation therapy (36–38).

#### 2.2.2 Pituitary adenoma/pituitary neuroendocrine tumor

Pituitary adenomas/pituitary neuroendocrine tumors are clonal neoplasms of the anterior pituitary hormone–producing cells. Only 5% of these tumors arise in the pediatric population.

##### 2.2.2.1 Clinical symptoms

Clinical symptoms largely depend upon the size and functional activity of the tumor. Tumors less than 1 cm in size that are slow growing and do not secrete pituitary hormones are usually diagnosed incidentally. Larger non-secreting tumors present with symptoms related to mass effect, including headache and visual disturbances from compression of the optic chiasm. Hormone-secreting tumors are usually diagnosed early on as patients present with hormone excess syndromes such as hyperprolactinemia, Cushing’s disease, or hyperthyroidism. Occasionally, these tumors undergo acute hemorrhagic necrosis (apoplexy) and patients present with severe headache, lethargy, and signs of increased intracranial pressure (39).

##### 2.2.2.2 Imaging

**Figure 3:**
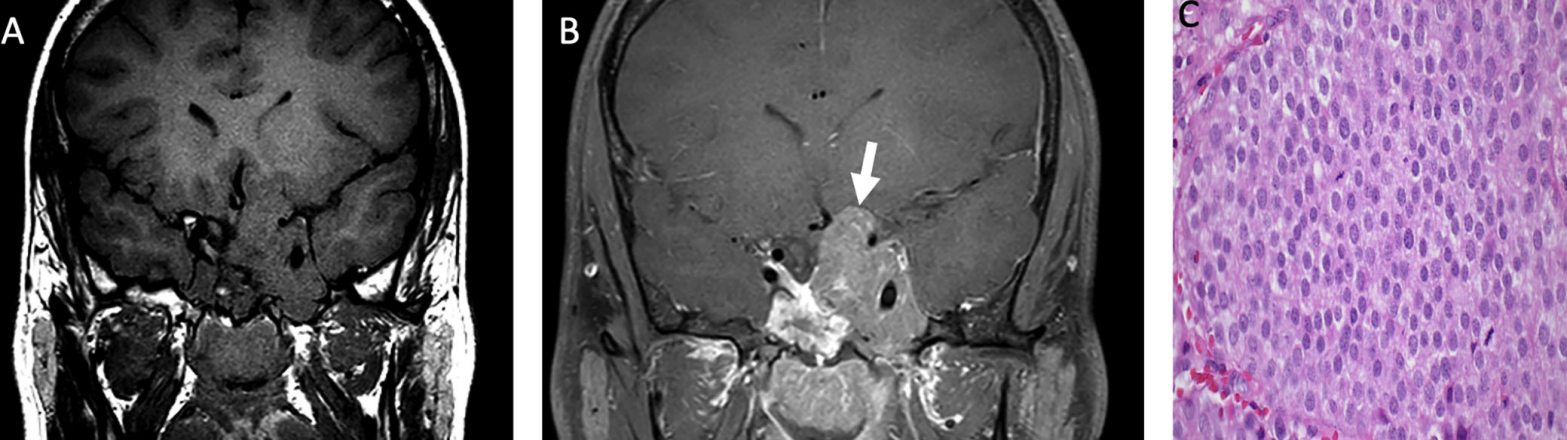
Obtained from a patient at St. Jude Children’s Research Hospital, Memphis, Tennessee. Coronal pre-contrast T1-weighted **(A)** and post-contrast T1-weighted **(B)** imaging demonstrating a T1 isointense, heterogeneously enhancing sellar/suprasellar mass invading the left greater than right cavernous sinus and the left hypothalamus (arrow). The histologic section **(C)** shows sheets of tumor cells with “salt-and-pepper” chromatin in the nuclei. There is a loss of the usual lobular architecture and mixed cell population of the normal adenohypophysis. Apparent mitotic activity can be found in biologically aggressive tumors.

Most pituitary adenomas/pituitary neuroendocrine tumors are hypointense on T1-weighted imaging, with variable degrees of enhancement on post-contrast images, relative to native pituitary tissue (**Figure 3**). A dynamic sequence is frequently needed to diagnose pituitary microadenoma (<1 cm of size) as the rate of gadolinium-based contrast accumulation within the tumor lags behind that of the normal pituitary gland. The T2 signal is variable: densely granulated tumors demonstrate T2 hypointensity, and sparsely granulated tumors demonstrate T2 hyperintensity. Pituitary macroadenomas may locally invade the hypothalamus, the sphenoid, and cavernous sinuses (39).

##### 2.2.2.3 Histopathology and molecular markers

On histopathology, pituitary adenomas demonstrate monomorphic cells arranged in diffuse, papillary, and trabecular arrangements. Cytologically, the cells may be acidophilic, basophilic, or chromophobic with densely or sparsely granulated cytoplasm and low mitotic activity. Molecular alterations in *GNAS* leading to upregulation of the cAMP/PKA pathway have been encountered in up to 40% of somatotroph adenomas, whereas mutations in *USP8* and *USP48* are identified in up to 50% of corticotroph adenomas (4). In addition, both functioning and non-functioning pituitary tumors have been encountered in a multitude of genetic syndromes, as described in **Table 2**.

**Table 2:** Inherited genetic susceptibility to pituitary tumors (4).

Genetic Disease	Genes involved	Pituitary tumors
Isolated pituitary tumors		
Familial isolated pituitary adenoma	*AIP* or unknown	Somatotroph, lactotroph, mammosomatotroph, and corticotroph tumors
X chromosome–linked acrogigantism	*GPR101*	Mammosomatotroph adenomas
Syndromes associated with pituitary tumors		
Multiple endocrine neoplasia type 1	*MEN1*	Nonfunctioning lactotroph, somatotroph, and corticotroph tumors Multiple or plurihormonal tumors Somatotroph or mammosomatotroph hyperplasia
Multiple endocrine neoplasia type 4	*CDKN1B*	Nonfunctioning somatotroph and corticotroph tumors
Carney complex	*PRKAR1A*	Somatotroph, lactotroph, and corticotroph tumors Mammosomatotroph or somatotroph hyperplasia
McCune-Albright syndrome	*GNAS*	Somatotroph, lactotroph, and corticotroph tumors Mammosomatotroph or somatotroph hyperplasia
Familial paraganglioma, pheochromocytoma, pituitary adenoma syndrome	*SDHA, SDHB, SDHC, SDHD*	Lactotroph, somatotroph, gonadotroph, and rarely corticotroph tumors
DICER1 syndrome	*DICER1*	Pituitary blastoma (mostly ACTH-secreting; rarely growth hormone– and prolactin-secreting)
Neurofibromatosis type 1	*NF1*	Corticotroph and somatotroph adenomas Pituitary duplication
Lynch syndrome	*MSH2*, *MSH6, MLH1*, *PMS2*	Corticotroph and lactotroph tumors
USP8-related syndrome	*USP8*	Corticotroph tumors
Tuberous sclerosis	*TSC1*, *TSC2*	Corticotroph tumors

##### 2.2.2.4 Management

In tumors that are small (>1 cm) in diameter, slow growing, and/or incidentally diagnosed, watchful waiting with serial MRIs is a commonly employed strategy. Endoscopic transsphenoidal resection of the tumor is the preferred treatment (40). Open craniotomy is required for complete resection of the larger tumor. Invasion of the cavernous sinus, which is rare, precludes gross total resection and might warrant radiation therapy for tumor control (41).

#### 2.2.3 Pituitary blastoma

Pituitary blastomas are exceptionally rare embryonal tumors composed of primitive blastemal cells, neuroendocrine cells, and Rathke’s pouch epithelium (42). They originate in the sellar region, with frequent invasion into the hypothalamus and cavernous sinuses. They usually occur in children younger than 2 years, with a slight female predilection (43).

##### 2.2.3.1 Clinical symptoms

Patients usually present with Cushing syndrome, with an elevated non-suppressible ACTH level (43). Ophthalmoplegia may occur due to tumor extension into the parasellar region (44).

##### 2.2.3.2 Imaging

Given the rarity of this entity, no hallmark imaging features have been described, with reported cases describing findings ranging from an enhancing solid mass limited to the sella to a mixed solid and cystic lesion with suprasellar/parasellar extension (45).

##### 2.2.3.3 Histopathology and molecular markers

Histologically, pituitary blastomas are composed of three cellular components: large anterior pituitary neuroendocrine cells, cuboidal or columnar primitive Rathke’s pouch epithelial cells, and small undifferentiated blastemal cells (46). Pituitary blastomas are linked to a germline and somatic variation in *DICER1*, a key gene in microRNA processing, and may thereby be associated with other *DICER1*-related tumors, namely, ovarian Sertoli-Leydig cell tumor, renal tumors, soft tissue sarcomas, and several thyroid proliferations (47–49).

##### 2.2.3.4 Management

Current treatment options include gross total resection when safe or subtotal resection with adjuvant chemotherapy or focal radiation therapy. Some of the commonly employed chemotherapy regimens include temozolomide alone or combination therapy with cyclophosphamide, vincristine, methotrexate, and carboplatin (49).

## 3 Tumors of the neurohypophysis

The neurohypophysis consists of the infundibulum and the pars nervosa (posterior lobe of the pituitary gland). The infundibulum consists of unmyelinated axons of neurosecretory cells, which originate in the hypothalamus and transport neuroendocrine hormones to the pars nervosa, where they are released into the systemic circulation via fenestrated sinusoidal capillaries. In the pediatric population, common neoplastic processes involving the infundibulum include Langerhans cell histiocytosis (LCH) and germ cell tumors. Tumors involving the pars nervosa, which are uncommon and include pituicytomas, gangliocytomas, and neurocytomas, will be discussed below.

### 3.1 Langerhans cell histiocytosis

LCH is characterized by an abnormal clonal proliferation of monocytes, macrophages, and dendritic cells.

#### 3.1.1 Clinical symptoms

LCH presents as a solitary bone lesion or single-system or multi-system involvement. Children between the ages of 0 and 15 years are affected, with a peak incidence between 1 and 4 years and a slight male predilection. Infiltration of the infundibulum and hypothalamus is encountered in up to 20% of patients with the multisystem form of LCH. Diabetes insipidus is the most common presenting clinical symptom. Symptoms related to anterior pituitary hormone deficiencies are less common.

#### 3.1.2 Imaging

**Figure 4:**
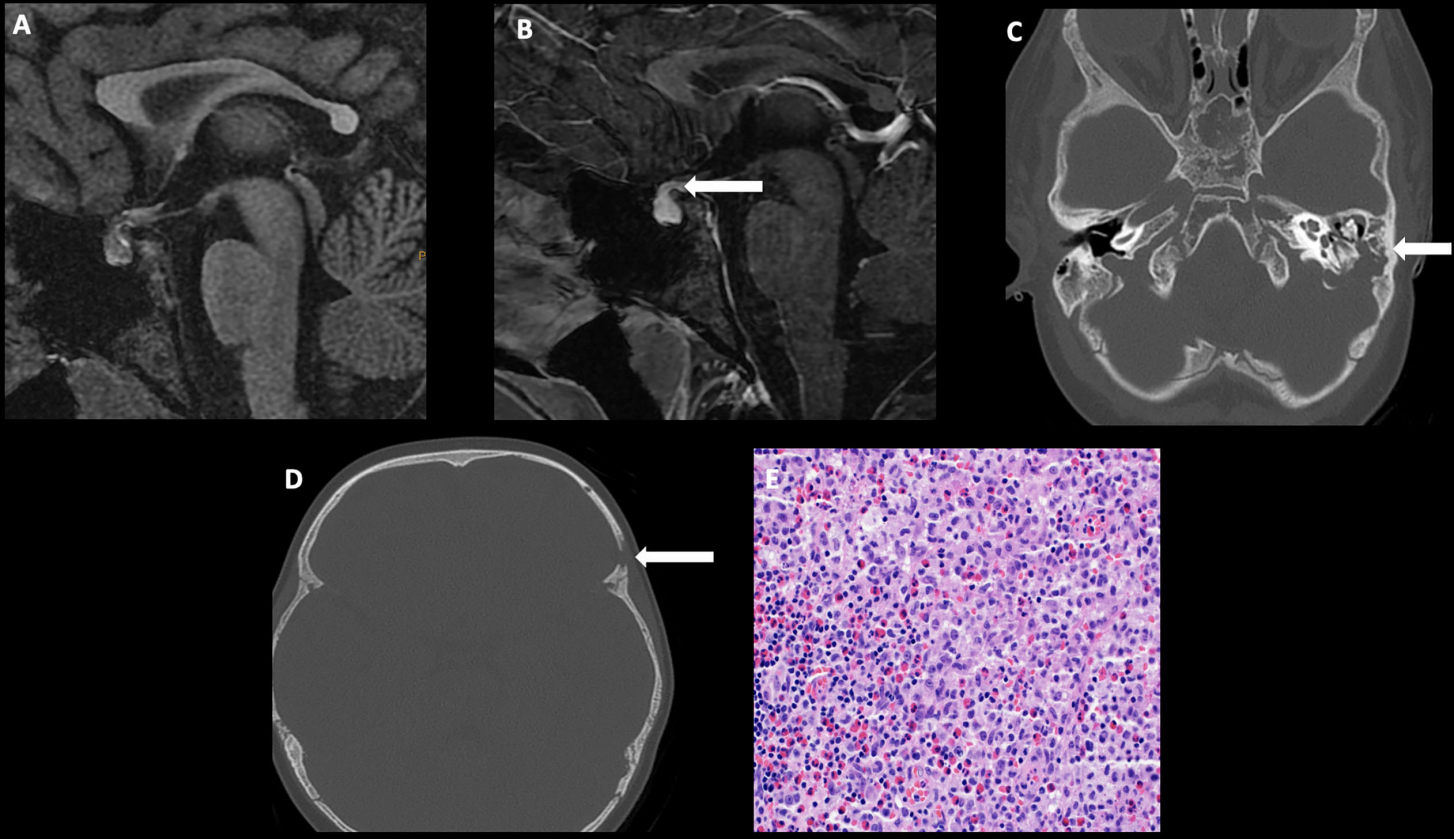
Obtained from a patient at St. Jude Children’s Research Hospital, Memphis, Tennessee. Sagittal T1 MRI images of pre- **(A)** and post- **(B)** contrast sequences demonstrating infundibular thickening >3 mm (arrow). Axial non-contrast CT of the temporal bone **(C)** demonstrating lytic-destructive change of the left mastoid air cells (arrow). **(D)** Axial CT image of the head, bone algorithm demonstrating a lytic lesion with a beveled edge in the left frontal calvarium (arrow). The histologic section **(E)** demonstrates numerous large and atypical tumor cells with abundant cytoplasm, prominent nucleoli, and irregular nuclear contour intermixed with many eosinophils and lymphocytes. The lesion can easily masquerade as an inflammatory or granulomatous process to inexperienced eyes.

Imaging often reveals nodular thickening and enhancement of the infundibulum, which measures more than 3.5 mm in transverse dimension and/or an enhancing suprasellar mass. Patients may have additional intracranial involvement with dural-based masses and lytic calvarial lesions preferentially involving the skull base and mastoid air cells (**Figure 4**) (50).

#### 3.1.3 Histopathology and molecular markers

Langerhans cells with variable reactive macrophages, lymphocytes, plasma cells, and eosinophils are characteristic histologic findings. The nuclei of Langerhans cells are slightly eccentric, ovoid, and reniform or convoluted, with linear grooves and inconspicuous nucleoli, with abundant pale to eosinophilic cytoplasm (51). The most frequent molecular alteration is *BRAF* p.V600E mutation encountered in approximately 50%–60% of cases (52). Other less frequent alterations include the *BRAF* p.V600D and *ARAF* mutations and *BRAF* fusions (53).

#### 3.1.4 Management

Treatment of LCH in the central nervous system (CNS) depends on both the extent and severity of the disease. For example, solitary calvarial and extra-axial LCH masses are treated with surgical resection, and more extensive parenchymal and infundibular/suprasellar LCH involvement is treated with chemotherapy. The most common first-line chemotherapy regimen for LCH with CNS involvement includes a combination of vinblastine and prednisone, with purine analogs, such as cytarabine and cladribine reserved for patients whose disease does not respond to first-line treatment. The knowledge of *BRAF* and other MAPK pathway mutations has led to the use of *BRAF* and MEK inhibitors in prospective clinical trials of histiocytosis, including a small proportion of participants who have CNS LCH. Therefore, molecular-targeted therapies represent a valuable resource for patients with refractory LCH of the CNS (51–53).

### 3.2 Germ cell tumors of the infundibulum/suprasellar region

Germ cell tumors of the CNS comprise a group of immunophenotypic homologs of gonadal and other extra-CNS germ cell neoplasms that share certain molecular features. This group includes pure germinomas and nongerminomatous germ cell tumors, a group comprising mature and immature teratomas, yolk sac tumors, embryonal carcinomas, choriocarcinomas, and mixed germ cell tumors (4). Approximately 80%–90% of these tumors arise in the midline; the pineal region is most commonly affected, followed by the infundibulum/hypothalamus (54). Germ cell tumors of the infundibulum/hypothalamus are more prevalent in Asia than in Europe or the United States, with peak incidences reported at 10–14 years of age, with a slight female predilection (55).

#### 3.2.1 Clinical symptoms

Clinically, these tumors present with symptoms secondary to mass effect, with visual disturbances due to impingement on the optic chiasm, diabetes insipidus and delayed growth, and sexual maturation due to disruption of the hypothalamic–pituitary axis, or headache and vomiting secondary to ventricular obstruction (55).

#### 3.2.2 Imaging

**Figure 5:**
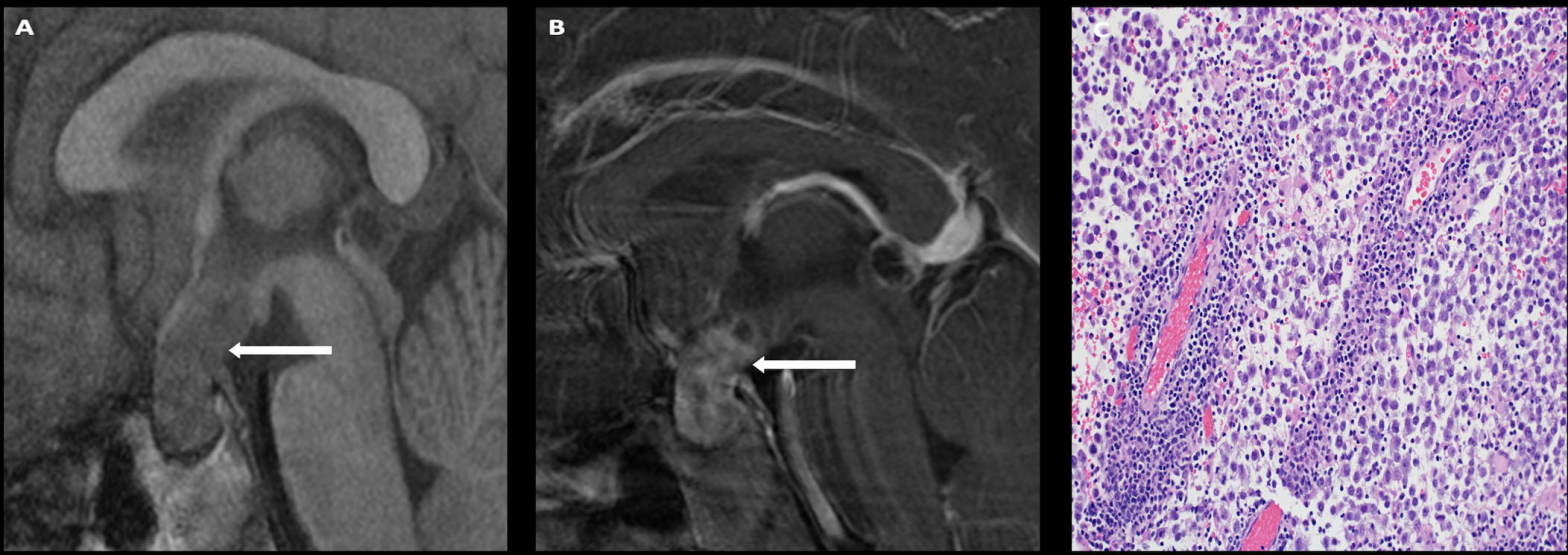
Obtained from a patient at St. Jude Children’s Research Hospital, Memphis, Tennessee. Sagittal T1 pre- **(A)** and post- **(B)** contrast sequences demonstrating a T1 isointense, avidly enhancing sellar/suprasellar mass (arrows). The native pituitary tissue and infundibulum are not distinctly identified. The histologic section **(C)** demonstrates numerous discohesive large tumor cells accompanied by many lymphocytes, a characteristic finding in germinoma.

**Figure 6:**
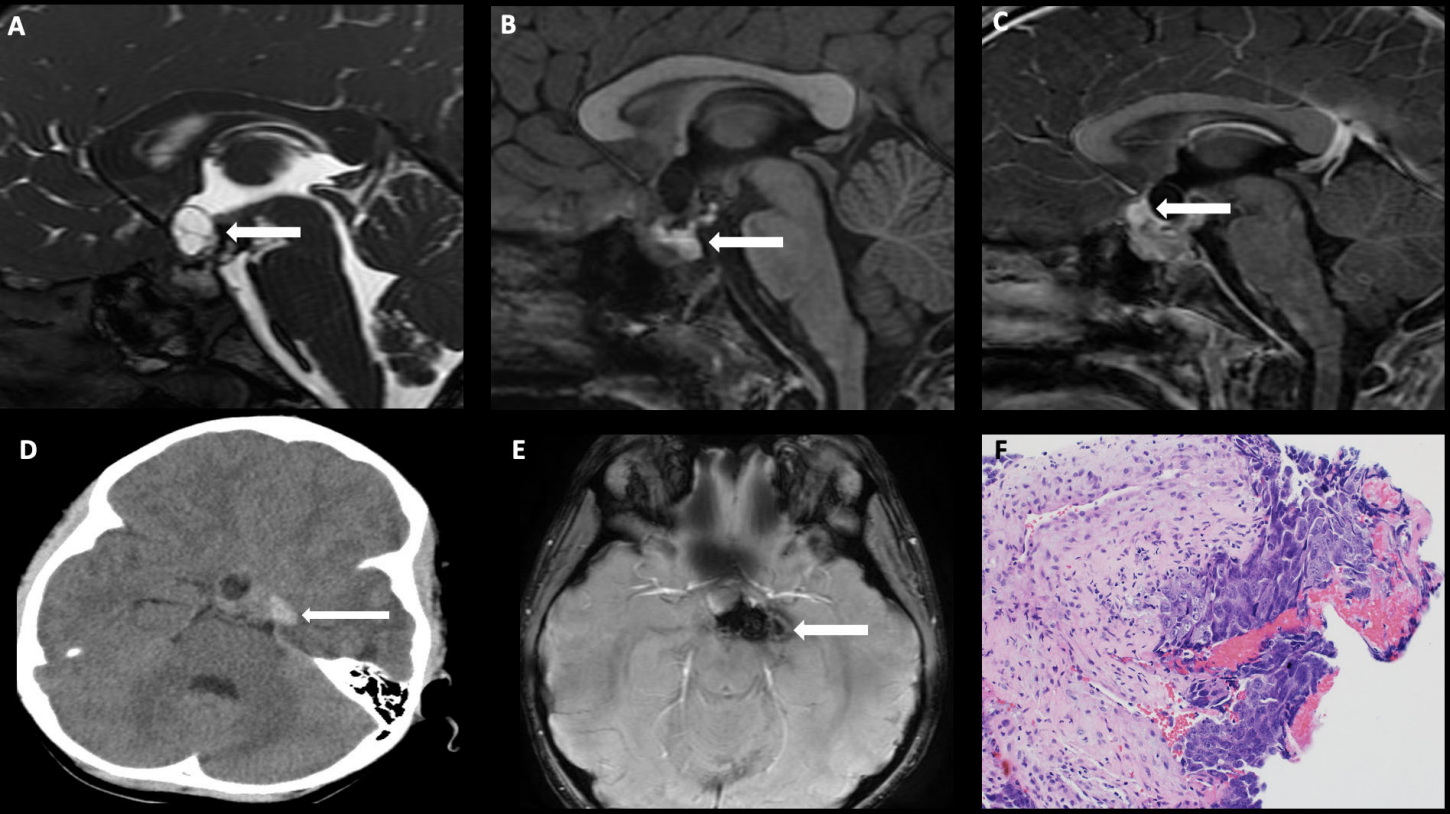
Obtained from a patient at St. Jude Children’s Research Hospital, Memphis, Tennessee. Sagittal FIESTA sequence demonstrating **(A)** a heterogeneous suprasellar mass with cystic components (arrow), **(B)** sagittal pre-contrast T1-weighted images demonstrating areas of intrinsic T1 hyperintensity compatible with blood products and/or proteinaceous debris (arrow), and **(C)** sagittal post-contrast T1-weighted images demonstrating an enhancing component in the anterior suprasellar cistern (arrow). **(D)** Axial non-contrast head CT demonstrating internal hemorrhage within the components to the left of midline (arrow), and **(E)** axial susceptibility-weighted imaging demonstrating a susceptibility artifact (arrow). The histologic section **(F)** shows embryonal carcinoma attached to the mesenchymal element of a teratoma. The large tumor cells in the embryonal carcinoma component have prominent nucleoli and basophilic cytoplasm.

On imaging, germinomas present as T1 isointense, T2 hyperintense, avidly enhancing masses involving the infundibular stalk and/or hypothalamus (**Figure 5**). On the other hand, nongerminomatous germ cell tumors are more heterogeneous in appearance, with areas of hemorrhage and intratumoral cysts identified in nearly all cases (**Figure 6**). Approximately 15% of germ cell tumors are metastatic at presentation, and imaging of the craniospinal axis along with CSF sampling is mandatory to assess for staging of disease. Although there is no dedicated staging system for intracranial germ cell tumors, most physicians utilize the TM/Chang system used for other brain tumors (56).

#### 3.2.3 Serum markers

The diagnosis involves careful interpretation of imaging findings in the context of laboratory markers, including beta-human chorionic gonadotropin (β-HCG), alpha fetoprotein (AFP), and placental alkaline phosphatase. The serum cutoff values for β-HCG vary by region, with more stringent European criteria suggesting serum levels as high as 50 mIU/ml for a diagnosis of pure germinoma and levels >50 mIU/ml for the diagnosis of nongerminomatous germ cell tumors. In contrast, the Children’s Oncology Group (COG) allows for serum levels as high as 100 mIU/ml for pure germinomas and >100 mIU/ml for nongerminomatous germ cell tumors. Serum levels of AFP >10 ng/ml and CSF levels of AFP >2 ng/ml, in the context of an intracranial mass, are considered diagnostic of nongerminomatous germ cell tumors. Levels of placental alkaline phosphatase are relatively sensitive and specific for a diagnosis of germinoma and are particularly helpful when serum and CSF levels of β-HCG and AFP are nondiagnostic. However, placental alkaline phosphatase alone is not considered a diagnostic criterion for germinoma, and a surgical biopsy may be needed particularly if the imaging is not diagnostic. In cases where the imaging and laboratory markers are diagnostic, a surgical biopsy is usually not warranted (57).

#### 3.2.4 Histopathology and molecular markers

Histopathological features vary depending on the type of germ cell tumor and are summarized in **Table 1**. Despite this heterogeneity, most germ cell tumors demonstrate common molecular alterations of the MAPK and/or AKT/mTOR pathways, with various degrees of mutations in the *KIT* gene, which ultimately inhibit programmed cell death of primordial germ cells in the CNS (4).

#### 3.2.5 Management

Treatment of pure germinomas has undergone significant reformation in recent years, with landmark clinical trials from COG and the International Society of Pediatric Oncology (SIOP) now advocating for the use of neoadjuvant chemotherapy, followed by maximal safe surgical resection and reduced-dose radiation for residual disease (58, 59). A response-based dose-reduction trial by COG (ACNS1123; Stratum 2) for patients with localized germinoma achieved a 3-year progression-free survival (PFS) of 94% ± 2.7% for patients treated with four courses of neoadjuvant carboplatin and etoposide, followed by gross total resection and 18 Gy whole-ventricular radiation, with a focal boost of 12 Gy to the tumor bed (58). In contrast, the SIOP CNS GCT II trial adopted 24 Gy whole-ventricular radiation in patients treated with neoadjuvant chemotherapy (alternating cycles of carboplatin + etoposide and ifosfamide + etoposide) followed by gross total resection (59). Treatment for nongerminomatous germ cell tumors is more intensive, given the higher rates of recurrence and poorer prognosis (60, 61). The SIOP CNS GCT-96 trial treated localized nongerminomatous germ cell tumors with four cycles of induction chemotherapy, followed by a focal boost of 54 Gy. The study achieved 5-year PFS and overall survival (OS) of 72% ± 4% and 82% ± 4%, respectively (60). On the other hand, the COG ACNS0122 study treated all patients with six cycles of induction chemotherapy, followed by 36 Gy craniospinal irradiation and a focal boost of 54 Gy to the tumor bed with excellent outcomes (61). Considering these data, the COG ACNS1123 Stratum 1 was developed, which used the same induction chemotherapy backbone as the ACNS0122 study but with a response-based radiation therapy plan. Per this plan, patients with a good response to chemotherapy were treated with 30.6 Gy whole-ventricular radiation with a focal boost of 23.4 Gy. Despite excellent results, this study was closed, as it met early stopping rules (62). For metastatic pure germinoma, the standard of care is 24 Gy craniospinal irradiation with a focal boost of 16 Gy to both primary and metastatic sites without chemotherapy, whereas metastatic nongerminomatous germ cell tumors are treated with four courses of chemotherapy, consisting of cisplatin, ifosfamide, and etoposide, followed by 30 Gy craniospinal irradiation and a focal boost of 16 Gy to the primary site (61, 63, 64).

### 3.3 Glial tumors of the pars nervosa

Pituicytoma, granular cell tumor of the sellar region, and spindle cell oncocytoma are a group of rare low-grade glial tumors that arise from the cells of the neurohypophysis or infundibulum. These group of tumors are likely representing variable manifestation of a single entity that overexpresses thyroid transcription factor-1 (TTF-1) (65, 66). These tumors typically present in adults with only a handful of case reports presenting in the first and second decades of life.

#### 3.3.1 Clinical symptoms

Visual disturbances due to suprasellar extension and mass effect on the optic chiasm is common presentation (67–70). These tumors do not produce hypothalamic or pituitary hormones (68).

#### 3.3.2 Imaging

On MRI, they demonstrate both solid and cystic components with early avid post-contrast enhancement, which persists into the venous phase (69).

#### 3.3.3 Histopathology and molecular markers

The tumors uniformly demonstrated spindle cells with a low mitotic index, and immunohistochemical stains were uniformly positive for vimentin, glial fibrillary acid protein (GFAP), and S-100 (4).

#### 3.3.4 Management

The hypervascular nature of these tumors and their proximity to the circle of Willis precluded gross total resection in these patients. These patients therefore underwent near total resection via an endoscopic transnasal, transsphenoidal, transcallosal, transseptal, or interforniceal approach with subsequent serial imaging to document stability (70).

### 3.4 Neuronal tumors of the pars nervosa

Other uncommon tumors of the pars nervosa include sellar neuronal tumors, such as gangliocytomas and neurocytomas (71). Gangliocytomas consist of larger, more mature neuronal elements and are more common in the adolescent and young adult population. Approximately 65% of gangliocytomas in the sellar region are associated with pituitary adenomas, and approximately 75% of them are associated with hypersecretion of a hypothalamic/pituitary hormone, most commonly GH-releasing hormone and ACTH-releasing hormone. These tumors most frequently present with endocrine abnormalities, such as acromegaly, Cushing disease, or syndrome of inappropriate antidiuretic hormone secretion confirmed by laboratory testing and immunohistochemical staining. On MRIs, these tumors are usually circumscribed, with homogeneous post-contrast enhancement (72, 73). Sellar neurocytomas, on the other hand, are extremely rare in the pediatric population, with only one case in an 8-year-old child reported in a case series of eight patients at a tertiary care institution (74). These tumors are WHO grade 2 lesions, with fibroblast growth factor receptor (FGFR) and isocitrate dehydrogenase wild type as their key molecular features (4). On imaging, these tumors are circumscribed, T1 isointense, T2 hyperintense, avidly enhancing lesions that can invade the adjacent suprasellar and parasellar structures. Surgical resection, either via a transcranial, transsphenoidal microscopic approach or an endoscopic endonasal approach, remains the mainstay of treatment, with adjuvant postoperative radiation reserved for patients with subtotal resection (74, 75).

## 4 Thalamic neoplasms

The thalamus consists of paired gray matter nuclei, which are located on either side of the third ventricle and are partitioned by a Y-shaped white matter structure, the internal medullary lamina (76). The subthalamus is an ovoid gray matter structure in the most caudal portion of the diencephalon, located lateral to the red nucleus and medial to the corticospinal tract. Tumors originating in the thalamus frequently extend into the subthalamic nuclei; therefore, these neoplasms will be discussed together (77). Thalamic tumors comprise 0.8%–5.2% of all pediatric brain tumors, with tumors of glial cell origin being the most common (78, 79). Less commonly, supratentorial ependymomas, embryonal tumors, and neuronal tumors may involve the thalamus and subthalamic nuclei. Surgical resection of tumors in this region is associated with significant postoperative morbidity and usually deferred for alternative treatments.

### 4.1 Pediatric-type diffuse astrocytoma

Pediatric-type diffuse astrocytomas are usually hemispheric tumors, which may occasionally involve the thalamus. They typically present in patients <19 years of age, with no definite sex predilection (80, 81).

#### 4.1.1 Clinical symptoms

Sensory or motor or mixed sensorimotor symptoms may be present in patients with predominant thalamic involvement. Patients with larger tumors can present with symptoms of raised intracranial pressure due to mass effect on the adjacent third ventricle and obstructive hydrocephalus.

#### 4.1.2 Imaging

These tumors demonstrate an infiltrative pattern, with intrinsic T1 isointensity to hypointensity, T2 heterogeneity or hyperintensity, with little to no contrast enhancement and no restricted diffusion. Differentiation of these tumors from its high-grade counterpart, diffuse midline glioma H3K27-altered, is not usually feasible on imaging. Surgical biopsy, which is usually performed under stereotactic guidance, is often needed for accurate histologic and molecular diagnosis (80).

#### 4.1.3 Histopathology and molecular markers

Histopathology usually demonstrates tumor cells with bland nuclei and rare mitotic activity, barely elevating the cell density of the infiltrated brain parenchyma. On immunohistochemical staining, tumor cells express GFAP with variable immunoreactivity for MAP2 and were negative for SOX10. In the most recent WHO classification, these tumors are classified as either diffuse low-grade glioma *MYB*- or *MYBL1*-altered and diffuse low-grade glioma MAPK pathway-altered. The latter category can be further subdivided into diffuse low-grade glioma *FGFR1*-altered (fusion or mutation) or diffuse low-grade glioma *BRAF* p.V600E-mutant (4, 82).

#### 4.1.4 Management

Treatment usually involves relief of obstructive hydrocephalus with a ventriculostomy or shunt, with surgical resection reserved for progressive disease. These tumors usually exhibit a benign clinical behavior, with 10-year PFS rates of 90% and 95% identified in one single-center study of 37 patients with *MYB* and *MYBL1*-altered tumors, respectively (81).

### 4.2 Pilocytic astrocytoma

Pilocytic astrocytomas are WHO grade 1 circumscribed astrocytic gliomas with variable proportions of bipolar hair-like pilocytic cells, compact and loose or myxoid regions, Rosenthal fibers, and eosinophilic granular bodies (4). A systematic review of 445 cases of thalamic gliomas in children revealed that pilocytic astrocytomas comprised approximately 33% of all of these tumors between the ages of 0 and 14 years (79).

#### 4.2.1 Clinical symptoms

Clinically, pilocytic astrocytomas in the thalamus present with symptoms secondary to elevated intracranial pressure and/or motor weakness (79).

#### 4.2.2 Imaging

**Figure 7:**
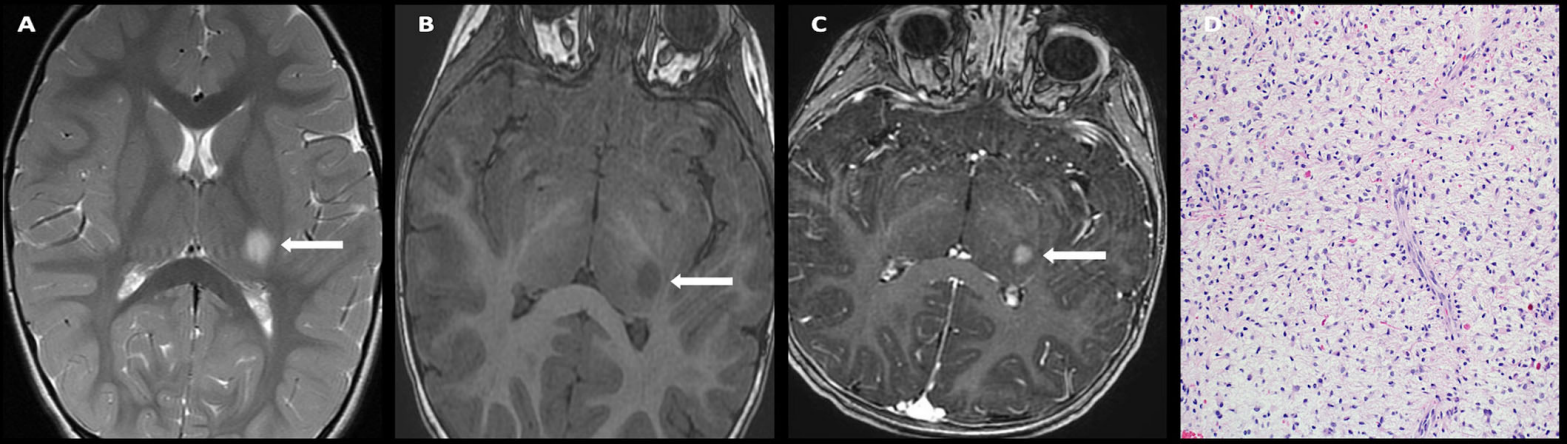
Obtained from a patient at St. Jude Children’s Research Hospital, Memphis, Tennessee. Axial T2 sequence **(A)** demonstrating a T2 hyperintense, circumscribed mass involving the left thalamus without significant mass effect. Axial pre-contrast T1-weighted **(B)** and post-contrast T1-weighted **(C)** images demonstrate a T1 hypointense, avidly enhancing mass. The histologic section **(D)** shows a piloid glial neoplasm with low levels of mild activity. Many eosinophilic Rosenthal fibers are present in the background. Prominent endothelial cells are a common finding in pilocytic astrocytomas.

Thalamic pilocytic astrocytomas are usually well-circumscribed T2 hyperintense tumors that can have both solid and cystic components (**Figure 7**) (83). Please refer to imaging of hypothalamic PAs for a detailed description.

#### 4.2.3 Histopathology and molecular markers

Pilocytic astrocytomas have low to moderate cellularity and include varying portions of piloid and oligodendrocyte-type cells. Multinucleated cells with horseshoe-shaped nuclear clusters (pennies-on-a-plate pattern) are often seen. Rosenthal fibers and eosinophilic granular bodies are common but vary in prominence. The most frequent molecular aberration is a chromosome 7q34 rearrangement resulting in a *KIAA1549:BRAF* fusion. Other aberrations include alternative *BRAF* fusions, *BRAF* mutations (especially p.V600E), and *NF1* and *FGFR1* mutations (4). Compared to the cerebellar pilocytic astrocytomas, the *KIAA1549:BRAF* fusion is less common in thalamic pilocytic astrocytomas.

#### 4.2.4 Management

Gross total resection is the treatment modality of choice and can often be achieved with preoperative diffusion tensor imaging to plan a corridor for approach, neuronavigation, tubular retractors to minimize adjacent white matter tract injury, and an exoscope for visualization (84). In cases where gross total resection cannot be achieved, targeted MEK inhibitor therapy is often employed, as most tumors harbor alterations in the MAPK genes (85).

### 4.3 Diffuse midline glioma, H3K27-altered

Diffuse midline H3K27-altered gliomas are WHO grade 4 pediatric high-grade gliomas that involve the brain stem, thalamus, and/or spinal cord. Thalamic involvement may be unilateral or bilateral; the unilateral pattern is more frequently seen in adolescents and young adults, and the bilateral pattern is more frequently seen in children. Thalamic diffuse midline gliomas represent 1%–5% of all pediatric brain tumors and as much as 25% of all pediatric thalamic tumors. The median age at presentation is 7–8 years with no clear sex predilection (86).

#### 4.3.1 Clinical symptoms

Common presentations of thalamic DMGs include symptoms related to intracranial hypertension and motor or sensory or mixed motor and sensory deficits (87).

#### 4.3.2 Imaging

**Figure 8:**
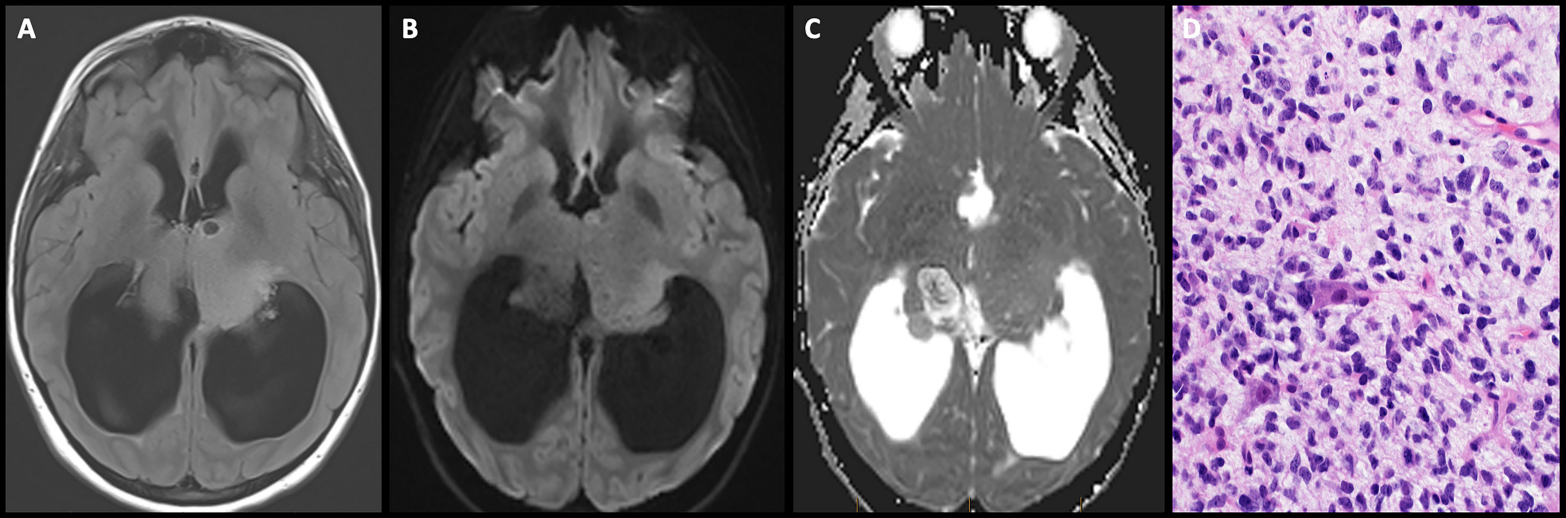
Obtained from a patient at St. Jude Children’s Research Hospital, Memphis, Tennessee. Axial FLAIR image **(A)** demonstrating a T2/FLAIR hyperintense infiltrative mass involving bilateral thalami with mass effect on the third ventricle and severe obstructive hydrocephalus. Axial diffusion-weighted **(B)** and apparent diffusion-coefficient maps **(C)** demonstrate no restricted diffusion. The histologic section **(D)** shows an infiltrative neoplasm with hyperchromatic nuclei and moderate nuclear pleomorphism. There are two large entrapped neurons.

Five distinct thalamic locations were observed in patients with thalamic DMGs enrolled in HERBY trial: thalamo-pulvinar, whole thalamus, uni-thalamic with diffuse spreads, anteromedial, and bithalamic in decreasing order of frequency (88). These tumors can be either well defined or infiltrative. They are usually T1 hypointense or isointense and T2 hyperintense with or without restricted diffusion (**Figure 8**). Peritumoral edema is characteristically absent in DMGs in contrast to other high-grade gliomas (89). Post-contrast enhancement is variable, with many tumors demonstrating little to no enhancement (88, 90). Radiographic necrosis can be present in up to 64% of tumors (88).

#### 4.3.3 Histopathology and molecular markers

Histopathology of these tumors demonstrates diffuse infiltration of the CNS parenchyma, without perivascular or perineuronal tropism. Most cells are small and monomorphic, but they can be polymorphous, showing astrocytic, piloid, oligodendroglial, giant cell, undifferentiated, or epithelioid cytology (4). The most common molecular abnormality of thalamic DMGs is an alteration in the 27th amino acid (lysine) on one of the Histone 3 (H3) isoforms, H3.1 or H3.2 or H3.3, and is present in up to 67% of thalamic DMGs (88). Other common molecular aberrations include H3-wild type with *EZHIP* overexpression or EGFR-mutant. The H3.1 or H3.2 K27-mutants are canonical sequence mutations, while H3.3 K-27 mutants are noncanonical in nature, both resulting in a widespread loss of H3 p. K28me3. In tumors with H3-wild-type molecular features, there is an overexpression of *EZHIP*, which acts as an endogenous H3 p.K28M mimic. *EGFR*-mutant diffuse midline glioma is characterized by abnormalities in the *EGFR* oncogene on chromosome band 7p11.2 (91).

#### 4.3.4 Management

Due to the unfavorable location, surgical resection is often not an option. Surgical biopsy is usually performed under stereotactic guidance for accurate molecular characterization. After diagnosis is confirmed, these tumors are treated with radiation and adjuvant chemotherapy. The prognosis of DMGs is poor, with a 2-year survival rate less than 10% (89).

### 4.4 Gangliogliomas

Gangliogliomas of the thalamus are uncommon in children; only a few cases have been reported in the literature (92, 93). These tumors are usually slow growing and most commonly arise during the first two decades of life with a slight male predilection (92).

#### 4.4.1 Clinical symptoms

Patients may present with sensory and/or motor deficits contralateral to the side of involvement (93).

#### 4.4.2 Imaging

Gangliogliomas demonstrate a mixed solid and cystic appearance, with variable post-contrast enhancement of the solid component. Calcifications may be present. At present, there are no imaging features associated with morphological features or tumor genotype (94).

#### 4.4.3 Histopathology and molecular markers

Histologically, glioneuronal tumors are biphasic, i.e., they consist of a variable mixture of neuronal and glial elements. The neuronal element consists of dysmorphic ganglion cells with abnormal clustering, a lack of cytoarchitectural organization, cytomegaly, perimembranous aggregation of Nissl substance, or binucleated forms. The glial element resembles a fibrillary astrocytoma, oligodendroglioma, or pilocytic astrocytoma. The most frequent molecular aberration is a *BRAF* p.V600E mutation, which is seen in 10%–60% of cases and activates the MAPK signaling pathway, which drives cellular proliferation. Gangliogliomas in the thalamus may exhibit concurrent *BRAF* p.V600E mutations and H3P.K28M mutations and are associated with an increased risk of local recurrence and lower overall survival (4).

#### 4.4.4 Management

Gross total resection is the treatment of choice, with targeted therapy with either MEK inhibitors (in cases of *BRAF* duplications or fusions) or *BRAF* inhibitors (in cases of *BRAF*V600E mutations) reserved for patients with subtotal resection. Radiation is also a viable treatment option in patients with subtotal resection. Surgical resection may be performed via a transtemporal, subtemporal, anterior interhemispheric transcallosal or posterior interhemispheric transtentorial approach (95).

### 4.5 Supratentorial ependymomas

Supratentorial ependymomas are glial tumors that arise from the primitive ependymal cells that line the ventricles. These tumors are located within the brain parenchyma and may be due to trapping of embryonic ependymal cells during the development of the cerebral hemispheres (4). In the pediatric population, supratentorial ependymomas comprise approximately 3% of all thalamic neoplasms and harbor either the *ZFTA* (C11orf95) or the *YAP1* fusion genes (4, 96). *ZFTA* fusion-positive ependymomas are more common, accounting for 66%–84% of all pediatric supratentorial ependymomas, with no specific gender predominance (96, 97). On the other hand, *YAP1* fusion-positive ependymomas account for only 6%–7.4% of all pediatric supratentorial ependymomas and are restricted to young children, with a male:female ratio of 0.3:1 (98).

#### 4.5.1 Clinical symptoms

Clinically, patients usually present with focal motor deficits, often accompanied by symptoms of elevated intracranial pressure such as headache and blurry vision. Altered sensorium may occur and is usually seen in the context of obstructive hydrocephalus. A few patients may present with an acute onset headache due to intratumoral and intraventricular hemorrhage (99).

Imaging: On imaging, both *ZFTA* and *YAP1* fusion-positive ependymomas have circumscribed margins, with solid and cystic components (99). The solid components often restrict diffusion, particularly in the *ZFTA* fusion-positive subtype, and demonstrate heterogeneous post-contrast enhancement. Intratumoral hemorrhage and peritumoral edema are more common in the *ZFTA* fusion-positive subtype (100).

#### 4.5.2 Histopathology and molecular markers

Histologically, supratentorial ependymomas may be WHO grade II or III tumors that demonstrate pseudorosettes and ependymal rosettes and comprise uniform small cells with round nuclei embedded in a fibrillary matrix. Both *ZFTA* and *YAP1* fusion-positive ependymomas are demarcated from adjacent brain, with small- to medium-sized round nuclei. True ependymal rosettes may be encountered in the *YAP1* fusion-positive subtype but are relatively rare in the *ZFTA* fusion-positive subtype. Molecularly, the *ZFTA* fusion-positive subtype is characterized by the fusion of the *ZFTA* gene with partner genes, mainly *RELA*, resulting in a pathological activation of NF-κB signaling. Homozygous deletions of *CDKN2A* indicate a disruption of cell cycle control in a subset of these tumors. In *YAP1* fusion-positive ependymomas, fusions of *YAP1* with *MAMLD1* or other partner genes are the principal oncogenic drivers of the disease through the recruitment of nuclear factor I (NFI) and TEA domain (TEAD) family domains (4).

#### 4.5.3 Management

Maximal safe surgical resection followed by focal radiation forms the mainstay of treatment for supratentorial ependymomas for patients over 12 months of age. The benefit of maintenance chemotherapy following radiation in this age group has been investigated by two phase III randomized clinical trials. The Children’s Oncology Group ACNS0831 trial evaluated the benefit of a 4-week maintenance chemotherapy regimen with vincristine, cisplatin, cyclophosphamide, and etoposide in newly diagnosed ependymoma patients over 12 months of age following maximal safe surgical resection and focal radiation. Patients were randomized to receive either focal radiation alone or radiation and maintenance chemotherapy post-resection. Their results at a median follow-up of 42.6 months suggested that patients who received any maintenance chemotherapy had a higher 3-year event-free survival as compared to those who did not receive any chemotherapy (p < 0.05). However, noncompliance rates were high in both the radiation and radiation + maintenance chemotherapy groups, and genomic testing is ongoing to determine which genetic subtypes may benefit the most from post-radiation maintenance chemotherapy (101). The International Society of Pediatric Oncology (SIOP) ependymoma II protocol is currently investigating the potential benefit of maintenance chemotherapy on stratum 1 in children over the age of 12 months treated with gross total resection and focal radiation by randomizing them to observation alone versus 16 cycles of maintenance chemotherapy with vincristine, etoposide, cyclophosphamide, and cisplatin. Stratum 2 of the protocol randomizes children over the age of 12 months of age with residual disease on imaging to induction chemotherapy with either vincristine, etoposide, and cyclophosphamide alone or with additional high-dose methotrexate. For patients with residual non-resectable disease following induction chemotherapy, patients will be further randomized to receive either focal radiation alone or with an additional 8 Gy boost to the tumor site (102).

In children below 12 months of age, chemotherapy is an integral component of adjuvant therapy, and radiation is typically delayed until the second year of life. Results from the SJYC07 trial demonstrated that four cycles of postoperative systemic chemotherapy followed by consolidative conformal focal radiation and 6 months of oral maintenance chemotherapy resulted in 4-year PFS rates of 72.6% ± 7.2% and overall survival rates of 92.6% ± 4.4%. Subtotal resection was associated with an inferior PFS rate at 4 years as compared to the gross total and near total resection groups (103).

## 5 Tumors of the pineal region

The epithalamus forms the posterior portion of the diencephalon and consists of the pineal gland, the habenular trigone, and the stria medullaris, a bundle of white matter axons connecting the hypothalamus to the habenular trigone. The pineal gland is a neuroendocrine organ involved in regulating the circadian rhythm. It is bounded superiorly by the splenium of the corpus callosum, inferiorly by the quadrigeminal plate cistern, and laterally by the thalamus. Tumors of the pineal gland account for approximately 3%–8% of all brain tumors in the pediatric population and are described in detail below (54).

### 5.1 Germ cell tumors of the pineal region

The most common pediatric pineal region tumors are germ cell tumors, which account for approximately 50%–75% of all pineal region tumors, with incidence peaks at 10–14 years of age and a clear male predominance for all histologic subtypes. These tumors are also more prevalent in Asian populations than in European or North American populations (54).

#### 5.1.1 Clinical symptoms

Patients with germ cell pineal tumors present with headache due to obstructive hydrocephalus caused by compression of the cerebral aqueduct and/or upward gaze palsy with loss of convergence (Parinaud syndrome), resulting from tumor invasion of the tectal plate. Preserved pupillary accommodation with impaired constriction (Argyll Robertson pupil) is also frequent (54).

#### 5.1.2 Imaging

these tumors are typically isointense to hypointense on T1-weighted imaging, isointense to hyperintense on T2-weighted imaging, with avid heterogeneous enhancement. These tumors usually restrict diffusion and may demonstrate susceptibility artifact secondary to calcification. Magnetic resonance spectroscopy of these tumors demonstrates an elevated choline/creatine ratio and a markedly elevated lipid/lactate peak (104).

#### 5.1.3 Histopathology and molecular markers

The histopathological subtypes and molecular features of these tumors have been elaborated in the section on infundibular/hypothalamic germ cell tumors and are described in **Table 3** (4).

**Table 3:** Histologic subtypes of CNS germ cell tumors (4).

Tumor type	Essential histologic criteria
Mature teratoma	Differentiation along at least two of the three somatic tissue lines (ectoderm, endoderm, mesoderm) Fully differentiated, adult-type histology (absence of fetal-type elements) Absence of other germ cell tumor components
Immature teratoma	Incomplete differentiation along at least two of the three somatic tissue lines Absence of other germ cell tumor components
Teratoma with somatic-type malignancy	Distinct histologic component with cytological features, architecture, mitotic activity Disorderly growth pattern expected of a sarcoma, carcinoma, or other defined type of somatic cancer in a mature or immature teratoma
Germ cell tumor	Large tumor cells Nuclear OCT4 and widespread membranous KIT (or podoplanin) Absence of CD30 expression Absence of AFP expression β-hCG immunoreactivity in syncytiotrophoblastic giant cells (for the specific diagnosis of germinoma with syncytiotrophoblastic elements) Absence of other germ cell tumor components (except syncytiotrophoblastic giant cells for the specific diagnosis of germinoma with syncytiotrophoblastic giant cells)
Embryonal carcinoma	Large epithelioid cells and CD30 and OCT4 expression Absent (or only focal) nonmembranous KIT expression Absence of β-hCG expression Absence of AFP expression Absence of other germ cell tumor components Cytokeratin expression is desirable
Yolk sac tumor	Epithelioid cells, with or without mesenchymal components Absence of other germ cell tumor components AFP expression Absent (or only focal) nonmembranous KIT expression Absent (or only focal) CD30 expression Absence of β-HCG expression
Choriocarcinoma	Syncytiotrophoblastic and cytotrophoblast elements but no other germ cell tumor components β-HCG expression Absence of KIT expression Absence of AFP expression Absence of OCT4 expression
Mixed germ cell tumors	A germ cell tumor with at least 2 distinct germ cell tumor subtypes

#### 5.1.4 Management

Treatment for pineal germ cell tumors parallels that of germ cell tumors of the infundibulum/suprasellar region. It involves a combination of neoadjuvant chemotherapy, surgical resection, and radiation therapy (58–63). In addition, because patients with pineal tumors often present with obstructive hydrocephalus, CSF-diversion procedures such as endoscopic third ventriculostomy (preferred method) or ventriculoperitoneal shunt placement (less-favored procedure due to the risk of peritoneal seeding) are performed. When CSF-diversion procedures are performed, surgical biopsies can be safely performed simultaneously, if indicated. Occasionally, these procedures can be avoided and a temporary ventriculostomy can be placed if chemotherapy is promptly instituted (104).

### 5.2 Pineocytoma

Pineocytoma is a well-differentiated WHO grade 1 pineal parenchymal tumor. This is a tumor of the adult but can present at any age group.

#### 5.2.1 Clinical symptoms

Smaller lesions are usually incidentally diagnosed on imaging. Larger lesions can present with hydrocephalus due to obstruction of the cerebral aqueduct.

#### 5.2.2 Imaging

The tumors are well circumscribed and may be uniformly solid or mixed solid and cystic. It is hypodense or isodense on CT and isointense to hypointense on T1-weighted imaging and hyperintense on T2-weighted imaging. The solid components enhance with intravenous contrast.

#### 5.2.3 Histopathology and molecular markers

Pineocytoma is a well-differentiated moderately cellular neoplasm of the pinealocytes with occasional rosette formation. No chromosomal aberration has been associated with this tumor.

#### 5.2.4 Management

Pineocytomas are treated with surgical resection with excellent prognosis if there is gross total resection (105).

### 5.3 Pineal parenchymal tumor of intermediate differentiation

Pineal parenchymal tumor of intermediate differentiation (PPTID) is a tumor of the pineal gland that demonstrates intermediate characteristics between pineocytoma and pineoblastoma. It is usually a tumor of adults but can present in pediatric patients (106, 107).

#### 5.3.1 Clinical symptoms

Symptoms largely dependent upon size and are similar to the pineocytomas as described above.

#### 5.3.2 Imaging

The imaging appearance is also similar to the pineocytomas. However, this tumor frequently demonstrates diffusion restriction due to hypercellularity. Invasion of the adjacent structure and leptomeningeal metastasis can be present.

#### 5.3.3 Histopathology and molecular markers

This tumor is characterized by moderate to high cellularity and can be either diffuse or lobulated. Small in-frame insertion of *KBTBD4* is recurrent and characteristic for PPTID.

#### 5.3.4 Management

Gross total resection is the surgical goal. Usually, this tumor is treated with adjuvant radiation therapy following surgical removal with or without additional adjuvant chemotherapy. Tumor recurrence and metastasis are common (107).

### 5.4 Pineoblastoma

Pineoblastomas account for approximately 35% of all pineal parenchymal tumors with a median age of presentation of 6 years and a slight female preponderance (108).

#### 5.4.1 Clinical symptoms

Clinically, they present with signs and symptoms due to increased intracranial pressure due to aqueductal obstruction, neuro-ophthalmological dysfunction (Parinaud syndrome), and brain stem or cerebellar dysfunction (109).

#### 5.4.2 Imaging

**Figure 9:**
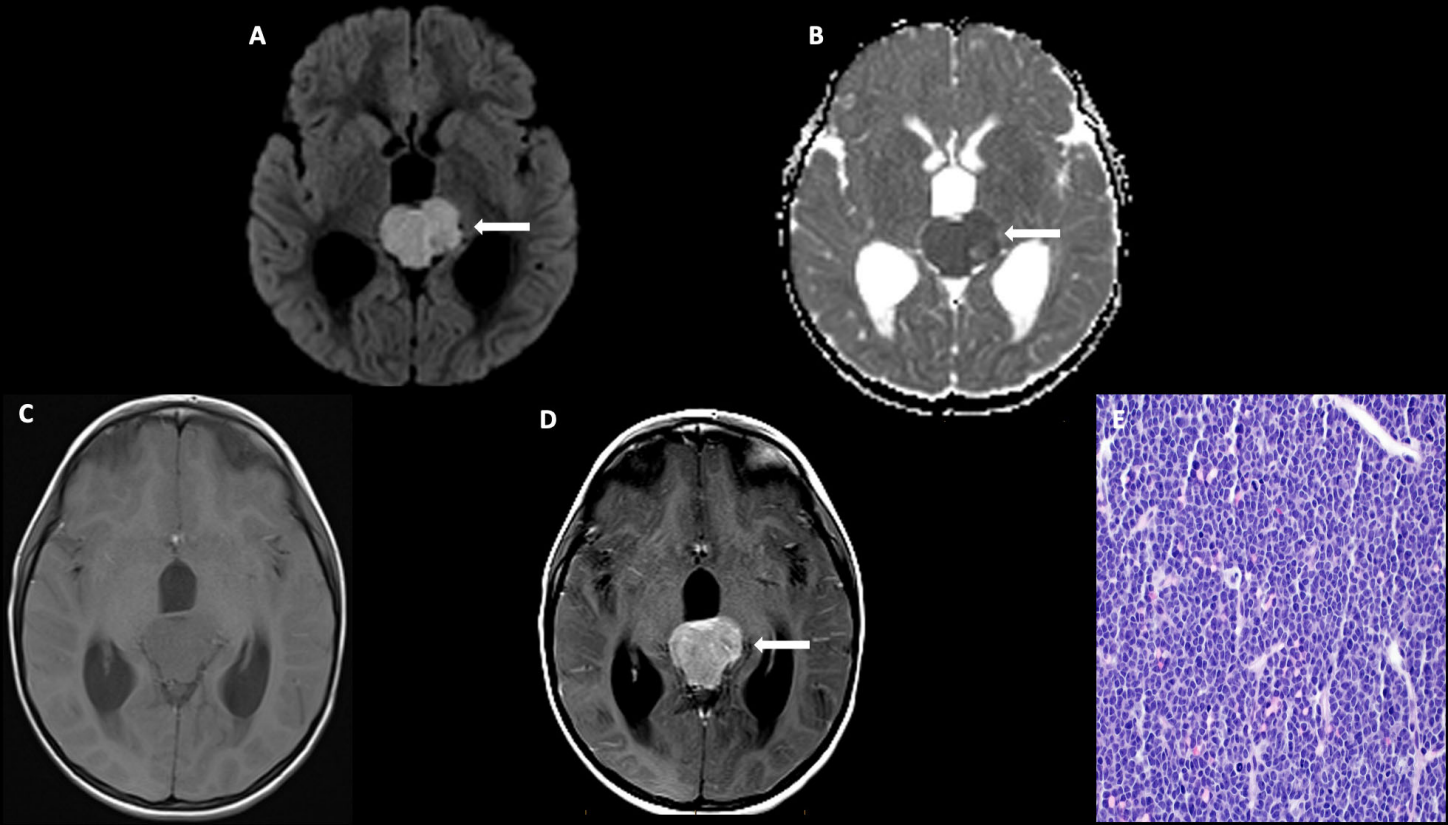
Obtained from a patient at St. Jude Children's Research Hospital, Memphis, Tennessee. **(A)** Axial diffusion-weighted imaging and **(B)** apparent diffusion coefficient map demonstrating a hypercellular pineal region mass causing obstructive hydrocephalus. **(C)** Axial pre-contrast and **(D)** post-contrast T1 images demonstrating avid enhancement within the mass. **(E)** The histologic section demonstrates a hypercellular embryonal tumor with readily apparent mitotic activity. The tumor cells show nuclear hyperchromasia and an inconspicuous amount of cytoplasm.

On imaging, they present as large masses with invasion of the surrounding structures, are hyperdense on CT, isointense on T1-weighted imaging with heterogeneous post-contrast enhancement (**Figure 9**). Peripheral calcifications are often seen. These tumors restrict diffusion due to hypercellularity. Craniospinal dissemination is observed in 25%–33% of tumors at presentation (110).

#### 5.4.3 Histopathology and molecular markers

On histopathology, pineoblastomas resemble other embryonal tumors of the CNS and are highly cellular with patternless sheets of densely packed small cells, irregular nuclei, and a high nuclear:cytoplasmic ratio. Pineoblastomas consist of distinct molecular subtypes, which impact overall prognosis and are summarized in **Table 4** (4). Treatment strategies vary by age, with children younger than 3 years treated with maximal safe surgical resection followed by intensive chemotherapy regimens, including high-dose chemotherapy with autologous stem cell rescue. A younger age is associated with poor 5-year progression-free and overall survival rates, with the largest pooled outcome study of 178 pineoblastoma cases reporting rates of 13.5% ± 5.1% and 16.2% ± 5.3%, respectively. The poorer prognosis in younger children has been linked to unfavorable molecular subtypes, surgical resection challenges, and the omission of radiation therapy (111).

**Table 4:** Clinical and molecular features of pineoblastoma (4).

Feature	Pineoblastoma subtypes			
	miRNA-processing-altered_ 1	miRNA-processing-altered_2	RB1-altered (pineal retinoblastoma)	MYC/FOXR2-activated
Median age (years)	8.5	11.6	2.1	1.3
Cancer predisposition	DICER1 syndrome	DICER1 syndrome	Hereditary retinoblastoma	None
Molecular features	CNAs; mutually exclusive mutations targeting *DICER1*, *DROSHA*, or *DGCR8*	CNAs; mutually exclusive mutations targeting *DICER1*, *DROSHA*, or *DGCR8*	–	–
Median OS (years)	10.4	Not reached	2.8	1.2
5-year OS (%)	68	100	29	23
5-year PFS (%)	54	93	27	13

Management: Safe surgical resection of pineoblastomas in children <3 years of age is limited by several technical and tumor-related factors. Firstly, neurosurgeons often encounter a difficulty in ideal head positioning in this age group due to suboptimal bone thickness and open sutures. Secondly, the hemorrhagic nature of these tumors coupled with the low blood volume of a failure to thrive infant precludes an aggressive resection. Third, the proximity of the pineal gland to the deep cerebral veins and the midbrain can lead to catastrophic neurologic sequelae in case of injury (112). Recent studies employing focal proton beam radiation in children <3 years with pineoblastoma have demonstrated improved outcomes. On the other hand, children older than 3 years are treated with maximal safe surgical resection, craniospinal irradiation with a focal boost, and intensive alkylator- and platinum-based chemotherapy regimens, with more favorable outcomes (113). Less aggressive molecular subtypes, higher rates of gross total resection, and the addition of radiation therapy are largely responsible for superior progression-free and overall survival rates in this age category. The presence of disseminated disease at presentation is associated with lower 5-year and PFS rates in older children but not in infants (114).

### 5.5 Papillary tumors of the pineal region

Papillary tumors of the pineal region (PTPR) are neuroepithelial tumors with both solid and papillary components. The reported age range of this tumor is between 1 and 71 years. In the pediatric population, the median age of presentation is approximately 11.6 years, with a slight female predilection (115–118). This entity was first described in 2003 and subsequently enlisted in the *WHO classification of CNS tumors* in 2007 as Grade 2 or 3 lesions (115).

#### 5.5.1 Clinical symptoms

Clinical presentations are similar to the other pineal parenchymal tumors. Imaging: On imaging, these tumors are heterogeneously enhancing, mixed solid and cystic masses that often obstruct the cerebral aqueduct, causing secondary hydrocephalus. Intrinsic T1 hyperintense proteinaceous secretions are also occasionally seen (115–118).

#### 5.5.2 Histopathology and molecular markers

On histopathology, these tumors demonstrate an epithelium-like appearance with papillary features and more densely cellular areas, often exhibiting ependymal-like differentiation (i.e., true rosettes and tubes). They also often express high levels of genes expressed in the subcommisural organ, including *SPDEF*, *ZFHX4*, *RFX3*, *TTR*, and *CALCA*. Recurrent chromosomal imbalances include losses from chromosome 10 and gains on chromosomes 4 and 9. Genetic alterations of *PTEN* have also been reported that constitutively activate the PI3K/akt/mTOR pathway and apoptotic dysregulation (119).

#### 5.5.3 Management

An effective treatment strategy has not been established for papillary tumors of the pineal region due to the scarcity of cases and the lack of randomized trials. However, gross total resection remains the mainstay of treatment in all age groups, with the largest multicenter study of 31 patients with papillary tumors reporting a 5-year overall survival of 73% in patients treated with gross total resection (120). In cases of subtotal resection, the propensity for tumor recurrence is high, and focal adjuvant radiation therapy with a boost to the tumor bed is recommended. Although currently, there is no definitive dose recommendation for adjuvant radiation therapy, 50 Gy is most frequently used (121, 122). The role of chemotherapy in papillary tumors remains controversial. Some studies have shown adjuvant chemotherapy regimens with carboplatin, vincristine, and temozolomide to be effective, whereas others have deemed them ineffective (123–125). Nevertheless, a systematic review in 2021 concluded that chemotherapy alone, both as a primary modality of treatment and as adjuvant therapy following resection, was not effective in preventing local recurrences in the pediatric population and should be used in conjunction with radiation (116).

## 6 Conclusions

Diencephalic tumors are complex midline tumors, and pediatric patients with these tumors usually present with symptoms secondary to mass effect on the hypothalamic–pituitary axis, optic nerves, or ventricles. Imaging plays a crucial role in localizing the primary tumor site and, occasionally, the cell of origin (e.g., glial, neuronal, primordial germ cell). Careful analysis of images often obviates the need for surgical biopsy and facilitates prompt institution of therapy. Surgical resection should be performed within maximal safe limits for most tumors, with neoadjuvant chemotherapy playing a key role in the preoperative management of germ cell tumors. Molecular analyses now enable the identification of potential targets in the treatment of certain entities, such as LCH and hypothalamic/optic pathway gliomas, and play a vital role in controlling disease. Although radiation therapy has several side effects, new lower-dose and conformal techniques have enabled higher treatment response rates with lower morbidity.

## Author contributions

SP: writing, editing. JC: Image collection, editing. IQ: editing. DL: image collection. AB: Conceptualization. All authors contributed to the article and approved the submitted version.
